# Multiplex Mediator Displacement Loop-Mediated Isothermal Amplification for Detection of *Treponema pallidum* and *Haemophilus ducreyi*

**DOI:** 10.3201/eid2602.190505

**Published:** 2020-02

**Authors:** Lisa Becherer, Sascha Knauf, Michael Marks, Simone Lueert, Sieghard Frischmann, Nadine Borst, Felix von Stetten, Sibauk Bieb, Yaw Adu-Sarkodie, Kingsley Asiedu, Oriol Mitjà, Mohammed Bakheit

**Affiliations:** University of Freiburg, Freiburg, Germany (L. Becherer, N. Borst, F. von Stetten);; Georg-August-University of Goettingen, Goettingen, Germany (S. Knauf);; German Primate Centre, Goettingen (S. Knauf, S. Lueert); London School of Hygiene & Tropical Medicine, London, UK (M. Marks);; Hospital for Tropical Diseases, London (M. Marks);; Mast Diagnostica GmbH, Reinfeld, Germany (S. Frischmann, M. Bakheit);; Hahn-Schickard, Freiburg, Germany (N. Borst, F. von Stetten);; Papua New Guinea Department of Health, Port Moresby, Papua New Guinea (S. Bieb);; Kwame Nkrumah University of Science and Technology, Kumasi, Ghana (Y. Adu-Sarkod);; World Health Organization, Geneva, Switzerland (K. Asiedu);; Lihir Medical Centre-International SOS, Newcrest Mining, Lihir Island, Papua New Guinea (O. Mitjà);; Fundacio Lluita contra la Sida-Hospital Universitari Germans Trias i Pujol, Badalona, Spain (O. Mitjà);; Barcelona Institute for Global Health-Hospital Clinic-University of Barcelona, Barcelona, Spain (O. Mitjà)

**Keywords:** Yaws, neglected tropical diseases, Treponema pallidum, Haemophilus ducreyi, loop-mediated isothermal amplification, LAMP, PCR, bacteria

## Abstract

Yaws, a neglected tropical disease caused by the bacterium *Treponema pallidum* subspecies *pertenue*, manifests as ulcerative skin lesions. Nucleic acid amplification tests, like loop-mediated isothermal amplification (LAMP), are versatile tools to distinguish yaws from infections that cause similar skin lesions, primarily *Haemophilus ducreyi*. We developed a novel molecular test to simultaneously detect *T. pallidum* and *H. ducreyi* based on mediator displacement LAMP. We validated the *T. pallidum* and *H. ducreyi* LAMP (TPHD-LAMP) by testing 293 clinical samples from patients with yaws-like lesions. Compared with quantitative PCR, the TPHD-LAMP demonstrated high sensitivity and specificity for *T. pallidum* (84.7% sensitivity, 95.7% specificity) and *H. ducreyi* (91.6% sensitivity, 84.8% specificity). This novel assay provided rapid molecular confirmation of *T. pallidum* and *H. ducreyi* DNA and might be suitable for use at the point of care. TPHD-LAMP could support yaws eradication by improving access to molecular diagnostic tests at the district hospital level.

Yaws, a neglected tropical disease caused by the bacterium *Treponema pallidum* subspecies *pertenue*, predominantly affects children living in low-income, rural communities of warm and humid regions (*1*). Clinical manifestations include lesions of the skin, bone, and cartilage, progressing to severe destructive lesions if left untreated (*2*). Manifestations of primary yaws include papillomas or ulcerative lesions; manifestations of secondary yaws include a wide range of rashes, often accompanied by bone and joint involvement (*2*). Currently, 15 countries in West and Central Africa, Southeast Asia, and the Pacific region are known to be yaws-endemic. The World Health Organization (WHO) released a yaws eradication strategy (the Morges strategy) in 2012 (*3*). The mainstay of the strategy is mass drug administration (MDA) with single-dose azithromycin in yaws-endemic communities, followed by routine surveillance and retreatment for 3–6 months until no cases remain (*3*).

Serologic tests, including the *T. pallidum* particle agglutination and rapid plasma reagin tests, remain the primary diagnostic tools for yaws (*2*). Newer point-of-care serologic tests have replaced traditional laboratory-based serologic assays in many settings (*4*–*7*). Despite their central role in yaws diagnosis, serologic assays have several limitations. First, treponemal serologic assays usually remain positive over a patient’s lifetime, and these tests cannot distinguish previous from current infection. Second, studies in Africa and in countries in the Pacific region have demonstrated that *Haemophilus ducreyi* causes cutaneous lesions similar to those observed in yaws (*8*–*11*). Persons with clinically suspicious lesions caused by *H. ducreyi* can have a reactive serologic test for yaws because of latent *T. pallidum* infection. Nucleic acid amplification tests (NAATs) can distinguish active yaws, involving a lesion with detectable *T. pallidum* DNA, from latent yaws, in which patients will have reactive serology without detectectable *T. pallidum* DNA from lesions. In addition, before seroconversion, a small proportion of patients with early active yaws will have a positive NAAT but negative serologic results.

NAATs could play a central role in yaws eradication efforts, particularly for diagnosis and surveillance after MDA in yaws-endemic areas (*12*). PCR has been standard for molecular diagnosis and has a high specificity and sensitivity for *T. pallidum* and *H. ducreyi*, but the process is time-consuming and requires expensive laboratory equipment. Most yaws-endemic countries have limited access to PCR to aid national yaws eradication programs. A point-of-care NAAT could provide reliable post-MDA molecular surveillance, as well as help in monitoring for azithromycin resistance. Loop-mediated isothermal amplification (LAMP) is an alternative for molecular diagnosis that might be more suitable than PCR as a point-of-care NAAT in resource-limited environments. LAMP has fast processing times and high specificity and can be performed on less expensive devices than those needed for PCR. 

Multiplex technologies, such as mediator displacement (MD) LAMP (*13*), have extended the usability of LAMP for simultaneous detection of >1 target and could be an efficient and cost-effective solution. MD detection uses an MD probe composed of a generic mediator attached to a generic overhang of a DNA target-specific sequence and a universal reporter molecule with a fluorophore and quencher for detection. We developed and validated a biplex MD LAMP assay to simultaneously identify *T. pallidum* and *H. ducreyi*. 

## Methods

### Participants

We obtained samples from larger trials conducted on Lihir Island (n = 57) and Karkar Island (n = 184), Papua New Guinea; and in Ghana (n = 52). Details of the studies in which the samples were collected are provided elsewhere (*14*,*15*). In brief, samples were collected as part of a randomized control trial comparing azithromycin doses of 30 mg/kg against doses of 20 mg/kg to treat patients in a pilot study for yaws elimination (*14*,*15*). Swabs were collected from persons with yaws-like ulcers and placed in AssayAssure Multilock (Sierra Molecular, https://sierramolecular.com) transport medium, then frozen at −20°C until transported to Mast Diagnostica GmbH laboratory in Reinfeld, Germany. DNA was extracted from the samples by using innuPREP MP Basic Kit A (Analytik Jena, https://www.analytik-jena.com) according to manufacturer’s instructions. Isolated DNA was kept frozen at −20°C until it was used for biplex *T. pallidum* and *H. ducreyi* LAMP (TPHD-LAMP), singleplex *T. pallidum* and *H. ducreyi* LAMP assays, and quantitative PCR (qPCR) testing.

### Ethics Approval

Participants, or parents or guardians of persons <18 years of age, provided written consent for inclusion in clinical surveys and etiologic studies. Children also provided assent when appropriate. The studies were approved by the National Medical Research Advisory Committee of the Papua New Guinea Ministry of Health (MRAC nos. 12.36 and 14.31), the Ghana Health Service (approval no. GHS 13/11/14), the London School of Hygiene & Tropical Medicine (approval no. 8832), and WHO (approval no. RPC720).

### TPHD-LAMP Assay

We devised the TPHD-LAMP assay on the basis of 2 previously published assays: a singleplex *T. pallidum* LAMP assay (*16*), which we modified by adding an MD probe; and a biplex LAMP assay of *T. pallidum* and *H. ducreyi* (*13*). TPHD-LAMP primers target the polymerase I (*polA*) gene of *T. pallidum* and the 16S ribosomal RNA (16S rRNA) of *H. ducreyi*. We further optimized the assays for improved functionality by redesigning primers and probes and modifying reagent concentrations (Appendix Tables 1–3). 

We performed a 2-step validation of the TPHD-LAMP assay. In the first step, we assessed the analytical sensitivity and specificity of the assay. In the second step, we used clinical samples collected in Ghana and Papua New Guinea to compare the performance of TPHD-LAMP against qPCR for individual targets. In a secondary analysis, we compared the performance of singleplex LAMP assays for each individual target against qPCR assays.

### Assessment of Analytical Performance

We determined the analytical limit of detection (LOD) for the TPHD-LAMP assay by using target sequences cloned into plasmids. We determined the LOD of each of the 2 components separately, as well as the LOD of the biplex TPHD-LAMP assay (Appendix). We varied the plasmid DNA concentrations between 3 × 10^1^ copies/reaction and 3 × 10^5^ copies/reaction in 8 replicates to reproduce the *Treponema* bacterial load in skin infections, which ranges from 10^2^–10^4^ copies/reaction (*17*). In addition, we tested the TPHD-LAMP in the presence of a high number of copies, 3 × 10^5^ copies/reaction, of *H. ducreyi* or *T. pallidum* in the presenece of a low number of copies of the second target to optimize each component and to simulate clinical samples that might contain both targets. We conducted primer titration experiments to minimize the preferential amplification of *H. ducreyi* DNA targets in persons with both infections. We estimated the LOD by counting the fraction of positive amplifications and performed probit regression analysis by using SPSS Statistics 25 (IBM, https://www.ibm.com).

We assessed the analytical specificity of the primer sets in silico by using ortholog target gene sequences from GenBank (Appendix Table 4) and found all primer sets were highly specific for *T. pallidum* and *H. ducreyi*. Based on these results, we tested the specificity of TPHD-LAMP in vitro against endemic pathogens associated with cutaneous ulcerative syndromes by using a panel of 13 organisms: *Escherichia coli*, *Klebsiella pneumoniae*, *Acinetobacter baumannii*, *Pseudomonas aeruginosa*, *Enterobacter cloacae*, *Salmonella enterica* (Paratyphi and Typhi), *Streptococcus pneumoniae*, *Streptococcus pyogenes*, *Staphylococcus aureus*, *Corynebacterium diphtheria*, *Corynebacterium ulcerans*, *Proteus mirabilis*, and *Enterococcus faecalis* (Appendix). We calculated interassay and intraassay variability of the TPHD-LAMP assay by using 3 batches of the TPHD-LAMP mix, prepared individually on 3 separate days and processed in different runs of 3 replicates per batch (Appendix).

### Clinical Performance of the TPHD-LAMP

We performed clinical validation by comparing the performance of the TPHD-LAMP and qPCR assays to identify *T. pallidum* and *H. ducreyi* in patient samples collected in Ghana and Papua New Guinea. TPHD-LAMP reactions (10 µL per assay) were composed of 1× RM MPM buffer (MAST Diagnostica GmbH, https://mast-group.com), 8 U Bst 2.0 WarmStart DNA Polymerase (New England Biolabs, https://www.neb.com), 0.05 µmol/L universal reporter, and MD primer mix (Appendix). We incubated primer mixes for 5 m at 70°C before LAMP to prevent nonspecific amplification initiated by primer dimerization. We performed real-time TPHD-LAMP reactions at 64°C in a Rotor-Gene Q (QIAGEN, https://www.qiagen.com) and acquired fluorescence signals every minute by using the Cy5-readout gain for *T. pallidum* and the FAM-readout gain for *H. ducreyi*. The singleplex LAMP reactions (10 µL per assay) using intercalating dye were composed of 1× RM MPM buffer, 8 U Bst 2.0 WarmStart DNA Polymerase, and 1 µL of 10x SYBR Green staining reagent, DNA free (AppliChem, https://www.applichem.com) and primer mix (Appendix Table 1). We also performed singleplex LAMP reactions in a Rotor-Gene Q at 63°C with the FAM-readout gain. We used a cutoff of 60 m for biplex TPHD-LAMP and singleplex LAMP assays and considered samples with amplification beyond 60 m negative.

For performance analyses, we compared the TPHD-LAMP assay against TaqMan qPCR assays targeting *polA* of *T. pallidum* (*18*) and an optimized TaqMan qPCR assay targeting the 16S rRNA gene of *H. ducreyi* on the same DNA extract (Appendix Table 4, Figure 1). The 16S rRNA gene has been previously used in qPCR assays to detect *H. ducreyi* (*19*). We ran all tests in duplicate and included positive controls and DNA-free negative controls in each run. We used an identical sample volume, 2.5 µL/reaction, for TPHD-LAMP, singleplex LAMP, and qPCR. For samples that tested negative by qPCR but positive by TPHD-LAMP, we repeated qPCR in a single reaction with higher sample volumes (3 µL) to identify true negative test results.

### Statistical Analysis

For clinical validation, we compared the sensitivity and specificity of the TPHD-LAMP assay against TaqMan qPCR assays. In a secondary analysis, we compared the performance of singleplex LAMP assays to qPCR. We performed all analysis by using R version 3.4.3 (https://www.R-project.org).

## Results

### Analytical Sensitivity and Specificity

The LOD for the TPHD-LAMP assay was 357 copies/reaction (95% CI 265–535 copies/reaction) for *T. pallidum* and 293 copies/reaction (95% CI 199–490 copies/reaction) for *H. ducreyi*. When we added the second target at the higher concentration of 3 × 10^5^ copies/reaction to simulate clinical samples from persons infected with both bacteria, the LOD increased to 808 copies/reaction (95% CI 550–2,128 copies/reaction) for *T. pallidum* and 622 copies/reaction (95% CI 415–1,687 copies/reaction) for *H. ducreyi* (Appendix Figure 2). The TPHD-LAMP assay was negative for all other pathogens tested within 60 m, demonstrating high analytical specificity (Appendix Figure 3). We observed a minimal interassay or intraassay variation (Appendix Figure 4).

### Validation of TPHD-LAMP in Clinical Samples

For clinical validation, we used a sample set consisting of 293 lesion swabs collected from patients with suspected *T. pallidum* infection. Samples were collected in Lihir Island (n = 57; 19.5%) and Karkar Island (n = 184; 62.8%), Papua New Guinea; and in Ghana (n = 52; 17.7%). A total of 184 (62.8%) cases were in male patients and 109 (37.2%) in female patients; the median age of case-patients was 10 years (interquartile range [IQR] 8–12 years).

Using qPCR, we detected *T. pallidum* in 59 (20.1%) samples, *H. ducreyi* in 155 (52.9%) samples, and *T. pallidum* and *H. ducreyi* co-infection in 19 (6.5%) samples. When tested by TPHD-LAMP, we detected *T. pallidum* in 60 (20.5%) samples and *H. ducreyi* in 163 (55.6%) samples. We detected both targets in 12 (4.1%) samples. Taking qPCR as the reference standard, the diagnostic sensitivity of the TPHD-LAMP assay for *T. pallidum* was 84.7% and the specificity was 95.7%. For *H. ducreyi*, the sensitivity of the TPHD-LAMP assay was 91.6% and the specificity was 84.8% (Table 1). Kappa coefficients (κ), ranging from 0.7 to 0.9 for the detection of *T. pallidum* and from 0.7 to 0.8 for *H. ducreyi*, show substantial to excellent agreement between qPCR and TPHD-LAMP. Moderate agreement between qPCR and TPHD-LAMP (κ = 0.5) also was demonstrated for the simultaneous detection of both targets. The median time to amplification of *T. pallidum* was 11 min (IQR 9–15 min) and the median time to amplification of *H. ducreyi* was 10 min (IQR 8–24 min).

**Table 1:** Comparison of clinical performance of biplex loop-mediated isothermal amplification for detection of *Treponema pallidum* and *Haemophilus ducreyi* (TPHD-LAMP) against singleplex TaqMan quantitative PCR*

Characteristics	Sample size	*Treponema pallidum*	*Haemophilus ducreyi*
Total samples, no.	293		
No. positive		60	163
Sensitivity, % (95% CI)		84.7 (72.5–92.4)	91.6 (85.8–95.3)
Specificity, % (95% CI)		95.7 (92.0–97.8)	84.8 (77.4–90.1)
Lesions containing a single pathogen†	195		
No. positive		48	151
Sensitivity, % (95% CI)		92.5 (78.5–98.0)	94.1 (88.4–97.2)
Specificity, % (95% CI)		95.7 (92.0–97.8)	84.8 (77.4–90.1)
Lesions containing both pathogens†	19		
No. positive		12	12
Sensitivity, % (95% CI)		68.4 (43.5–86.4)	73.7 (48.6–89.9)
Specificity, % (95% CI)		NA	NA
Samples from Lihir Island, no.	57		
No. positive		21	13
Sensitivity, % (95% CI)		90.5 (68.2–98.3)	76.5 (50.0–92.2)
Specificity, % (95% CI)		94.4 (80.0–99.0)	100.0 (89.1–100)
Samples from Karkar Island, no.	184		
No. positive		33	119
Sensitivity, % (95% CI)		78.1 (59.6–90.1)	94.2 (87.5–97.7)
Specificity, % (95% CI)		94.7 (89.5–97.5)	74.7 (63.4–83.5)
Samples from Ghana, no.	52		
No. positive		6	31
Sensitivity, % (95% CI)		100.0 (51.7–100)	90.9 (75.5–97.6)
Specificity, % (95% CI)		100.0 (90.4–100)	94.7 (71.9–99.7)

*NA, not applicable. †Determined by quantitative PCR.

For samples in which only 1 organism was detected by qPCR, the sensitivity of the TPHD-LAMP assay was higher for both *T. pallidum* (92.5%) and *H. ducreyi* (94.1%) than for samples with both organisms confirmed by qPCR. For samples confirmed to contain both bacteria by qPCR, sensitivity for *T. pallidum* was 68.4% (p = 0.048) and sensitivity for *H. ducreyi* was 73.7% (p = 0.01) (Table 1).

Using qPCR as the reference standard, the singleplex *T. pallidum* LAMP assay had a sensitivity of 78.0% and specificity of 97.9%; for the singleplex *H. ducreyi* LAMP assay the sensitivity was 91.0% and specificity was 75.3% (Table 2). We did not see a noticeable variation in the performance of the biplex TPHD-LAMP and singleplex LAMP assays between locations from which samples were collected (Tables 1 and 2).

**Table 2:** Comparison of clinical performance of singleplex loop-mediated isothermal amplification for detection of *Treponema pallidum* and *Haemophilus ducreyi* against singleplex TaqMan quantitative PCR*

Characteristics	Sample size	*Treponema pallidum*	*Haemophilus ducreyi*
Total samples, no.	293		
No. positive		51	175
Sensitivity, % (95% CI)		78.0 (64.9–87.3)	91.0 (85.0–94.8)
Specificity, % (95% CI)		97.9 (94.8–99.2)	75.3 (67.2–82.1)
Lesions containing a single pathogen†	195		
No. positive		34	158
Sensitivity, % (95% CI)		82.5 (66.6–92.1)	92.6 (86.5–96.2)
Specificity, % (95% CI)		97.9 (94.8–99.2)	75.4 (67.2–82.1)
Lesions containing both pathogens†	19		
No. positive samples		17	17
Sensitivity, % (95% CI)		68.4 (43.5–86.4)	78.9 (53.9–93.0)
Specificity, % (95% CI)		NA	NA

*NA, not applicable. †Determined by quantitative PCR.

## Discussion

We provide data demonstrating a high analytical performance of a multiplex LAMP assay for *T. pallidum* and *H. ducreyi* and a high sensitivity and specificity comparable to qPCR. The TPHD-LAMP assay also performed better than singleplex LAMP assays, likely reflecting better performance of the MD technology used in the biplex LAMP compared with standard intercalating dyes used in singleplex LAMP assays. 

The LOD of the TPHD-LAMP assay was 300 copies/reaction for both targets, which is comparable to qPCR, which has standard reproducibitly in a range of 10^1^–10^6^ copies/reaction. The LOD increased to ≈600 copies/reaction in samples that contained both targets, which is consistent with our clinical validation of the TPHD-LAMP; sensitivity for both bacteria was slightly higher when samples contained only a single target. Kappa coefficients confirmed substantial agreement (κ>0.7) for the individual targets and moderate agreement (κ = 0.5) for simultaneous detection of both targets in a sample. 

Detection of *T. pallidum* is the programmatic priority, but detection of *H. ducreyi* is beneficial for clinical management of patients with suspected yaws. The median time to amplification was <15 m for both *T. pallidum* and *H. ducreyi*, indicating the TPHD-LAMP assay could provide rapid, molecular confirmation of the presence of *T. pallidum* or *H. ducreyi*. Further optimization of the assay to enhance the performance of the *T. pallidum* component, particularly in the context of coinfection, will be required to ensure cases of yaws are not missed.

Implementing qPCR at the point of care is operationally challenging because it requires relatively expensive equipment, in particular thermocyclers, which can cost up to 10 times as much as a tubescanner capable of performing the TPHD-LAMP assay. Because qPCR is available only in a limited number of national and international reference laboratories, TPHD-LAMP might be an alternative molecular test to support expansion of yaws eradication activities. We did not conduct a cost-effectiveness analysis of the TPHD-LAMP assay, but such an assessment should consider equipment costs, cost per assay, and the relative performance of each assay to assess the cost per case diagnosed. However, our data suggest that the TPHD-LAMP assay might be a cost-saving alternative to qPCR, especially at the point of care.

Our study had some limitations. We tested samples from only 2 geographic regions for clinical validation of the TPHD-LAMP. Primer binding site mutations have affected the performance of other diagnostic assays for *T. pallidum* strains. Although we selected conserved genomic regions when designing the TPHD-LAMP primers, further experimental validation of the TPHD-LAMP assay with samples from a broader range of settings is needed. We conducted clinical validation of the assay in a controlled laboratory setting, but conditions at the point of care, including temperature, humidity, and a range of other environmental factors, might affect reagents in storage and in performing assays. Further optimization, including freeze-dried reagents in combination with dried oligonucleotides, might improve robustness and facilitate rollout of the assay in yaws-endemic countries.

In yaws-endemic countries, clinical manifestations combined with serologic tests are still the standard tool for the clinical management of yaws, but serologic tests have limitations and molecular assays are needed to support WHO yaws eradication efforts (*12*). Molecular assays also can detect mutations in the 23S RNA gene associated with azithromycin resistance (*15*,*20*,*21*), which is essential to monitor for drug resistance as yaws eradication efforts expand. qPCR is the most common NAAT currently available but remains restricted to a small number of laboratories in yaws-endemic countries. MD LAMP could facilitate surveillance for resistance and we plan further studies to evaluate a modified TPHD-LAMP assay for this purpose. Further, multicountry evaluations are warranted to assess performance of the assay when deployed in yaws-endemic countries and to assess the role the test could play in support of national yaws eradication programs. Nonetheless, the performance characteristics of the TPHD-LAMP suggest it has the potential to increase access to molecular diagnosis of yaws, especially at the point of care. 

Appendix Additional information on biplex mediator displacement loop-mediated isothermal amplification for detection of *Treponema pallidum* and *Haemophilus ducreyi* in clinical samples.

## References

[1] Marks M, Solomon AW, Mabey DC. Endemic treponemal diseases. Trans R Soc Trop Med Hyg. 2014;108:601–7. 10.1093/trstmh/tru128 25157125 PMC4162659

[2] Mitjà O, Asiedu K, Mabey D. Yaws. Lancet. 2013;381:763–73. 10.1016/S0140-6736(12)62130-8 23415015

[3] The World Health Organization. Eradication of yaws—the Morges strategy. Wkly Epidemiol Rec. 2012;87:189–94. 24340400

[4] Ayove T, Houniei W, Wangnapi R, Bieb SV, Kazadi W, Luke L-N, et al. Sensitivity and specificity of a rapid point-of-care test for active yaws: a comparative study. Lancet Glob Health. 2014;2:e415–21. 10.1016/S2214-109X(14)70231-1 25103395

[5] Marks M, Goncalves A, Vahi V, Sokana O, Puiahi E, Zhang Z, et al. Evaluation of a rapid diagnostic test for yaws infection in a community surveillance setting. PLoS Negl Trop Dis. 2014;8:e3156. 10.1371/journal.pntd.0003156 25211018 PMC4161315

[6] Fitzpatrick C, Asiedu K, Sands A, Gonzalez Pena T, Marks M, Mitja O, et al. The cost and cost-effectiveness of rapid testing strategies for yaws diagnosis and surveillance. PLoS Negl Trop Dis. 2017;11:e0005985. 10.1371/journal.pntd.0005985 29073145 PMC5658197

[7] Marks M, Yin Y-P, Chen X-S, Castro A, Causer L, Guy R, et al. Metaanalysis of the performance of a combined treponemal and nontreponemal rapid diagnostic test for syphilis and yaws. Clin Infect Dis. 2016;63:627–33. 10.1093/cid/ciw348 27217216 PMC4981758

[8] González-Beiras C, Marks M, Chen CY, Roberts S, Mitjà O. Epidemiology of *Haemophilus ducreyi* infections. Emerg Infect Dis. 2016;22:1–8. 10.3201/eid2201.150425 26694983 PMC4696685

[9] Marks M, Chi K-H, Vahi V, Pillay A, Sokana O, Pavluck A, et al. *Haemophilus ducreyi* associated with skin ulcers among children, Solomon Islands. Emerg Infect Dis. 2014;20:1705–7. 10.3201/eid2010.140573 25271477 PMC4193279

[10] Mitjà O, Lukehart SA, Pokowas G, Moses P, Kapa A, Godornes C, et al. *Haemophilus ducreyi* as a cause of skin ulcers in children from a yaws-endemic area of Papua New Guinea: a prospective cohort study. Lancet Glob Health. 2014;2:e235–41. 10.1016/S2214-109X(14)70019-1 25103064

[11] Ghinai R, El-Duah P, Chi K-H, Pillay A, Solomon AW, Bailey RL, et al. A cross-sectional study of ‘yaws’ in districts of Ghana which have previously undertaken azithromycin mass drug administration for trachoma control. PLoS Negl Trop Dis. 2015;9:e0003496. 10.1371/journal.pntd.0003496 25632942 PMC4310597

[12] Marks M, Mitjà O, Vestergaard LS, Pillay A, Knauf S, Chen C-Y, et al. Challenges and key research questions for yaws eradication. Lancet Infect Dis. 2015;15:1220–5. 10.1016/S1473-3099(15)00136-X 26362174 PMC4668588

[13] Becherer L, Bakheit M, Frischmann S, Stinco S, Borst N, Zengerle R, et al. Simplified real-time multiplex detection of loop-mediated isothermal amplification using novel mediator displacement probes with universal reporters. Anal Chem. 2018;90:4741–8. 10.1021/acs.analchem.7b05371 29508609

[14] Marks M, Mitjà O, Bottomley C, Kwakye C, Houinei W, Bauri M, et al.; study team. Comparative efficacy of low-dose versus standard-dose azithromycin for patients with yaws: a randomised non-inferiority trial in Ghana and Papua New Guinea. Lancet Glob Health. 2018;6:e401–10. 10.1016/S2214-109X(18)30023-8 29456191 PMC7116878

[15] Mitjà O, Godornes C, Houinei W, Kapa A, Paru R, Abel H, et al. Re-emergence of yaws after single mass azithromycin treatment followed by targeted treatment: a longitudinal study. Lancet. 2018;391:1599–607. 10.1016/S0140-6736(18)30204-6 29428183 PMC5920722

[16] Knauf S, Lüert S, Šmajs D, Strouhal M, Chuma IS, Frischmann S, et al. Gene target selection for loop-mediated isothermal amplification for rapid discrimination of *Treponema pallidum* subspecies. PLoS Negl Trop Dis. 2018;12:e0006396. 10.1371/journal.pntd.0006396 29649256 PMC5978989

[17] Tipple C, Hanna MOF, Hill S, Daniel J, Goldmeier D, McClure MO, et al. Getting the measure of syphilis: qPCR to better understand early infection. Sex Transm Infect. 2011;87:479–85. 10.1136/sti.2011.049494 21752804 PMC3252622

[18] Chen C-Y, Chi K-H, George RW, Cox DL, Srivastava A, Rui Silva M, et al. Diagnosis of gastric syphilis by direct immunofluorescence staining and real-time PCR testing. J Clin Microbiol. 2006;44:3452–6. 10.1128/JCM.00721-06 16954299 PMC1594693

[19] Orle KA, Gates CA, Martin DH, Body BA, Weiss JB. Simultaneous PCR detection of *Haemophilus ducreyi, Treponema pallidum*, and herpes simplex virus types 1 and 2 from genital ulcers. J Clin Microbiol. 1996;34:49–54. 8748271 10.1128/jcm.34.1.49-54.1996 PMC228728

[20] Chen C-Y, Chi K-H, Pillay A, Nachamkin E, Su JR, Ballard RC. Detection of the A2058G and A2059G 23S rRNA gene point mutations associated with azithromycin resistance in *Treponema pallidum* by use of a TaqMan real-time multiplex PCR assay. J Clin Microbiol. 2013;51:908–13. 10.1128/JCM.02770-12 23284026 PMC3592075

[21] Lukehart SA, Godornes C, Molini BJ, Sonnett P, Hopkins S, Mulcahy F, et al. Macrolide resistance in *Treponema pallidum* in the United States and Ireland. N Engl J Med. 2004;351:154–8. 10.1056/NEJMoa040216 15247355

