# Supplementary material for: Multiplex Mediator Displacement Loop-Mediated Isothermal Amplification for Detection of Treponema pallidum and Haemophilus ducreyi
Source: Emerg Infect Dis. 2020 Feb;26(2):282–8. doi: 10.3201/eid2602.190505 (PMC6986840; doi:10.3201/eid2602.190505)
Supplement: Appendix — Additional information on biplex mediator displacement loop-mediated isothermal amplification for detection of Treponema pallidum and Haemophilus ducreyi in clinical samples. [file 19-0505-Techapp-s1.pdf]

Article DOI: https://doi.org/10.3201/eid2602.190505

Multiplex Mediator Displacement Loop-Mediated Isothermal Amplification for Detection of *Treponema pallidum* and *Haemophilus ducreyi*

Appendix

Assay Optimization

Oligonucleotide and Primer Design

For the *Treponema pallidum* and *Haemophilus ducreyi* loop-mediated isothermal amplification (TPHD-LAMP) assay, we redesigned and further optimized oligonucleotides previously described by Knauf et al. (*1*) for detecting *T. pallidum* and by Becherer et al. (*2*) for detecting *H. ducreyi* to improve assay performance. In brief, we designed primers from GenBank sequences of the 16S ribosomal RNA gene of *H. ducreyi* and the *polA* gene of *T. pallidum* using PrimerExplorer V5 (Fujitsu, http://primerexplorer.jp) software.

Mediator displacement (MD) LAMP (*2*) for the simultaneous detection of multiple targets requires MD probes and fluorogenic universal reporter (UR) molecules for signal generation. An MD probe is comprised of a universal mediator (Med) combined with a modified Loop F (LF) or Loop B (LB) primer. The modified primer (LB/LF_Medc) contains target-specific primer sequences (LF or LB) at the 3′-end and a universal sequence at the 5′-end (Medc) that is complementary to the mediator. We designed MD probes in silico with Visual OMP version 7.8.42.0 (DNA Software, https://www.dnasoftware.com) software, as described previously (*2*), and used URs described by Lehnert et al. (*4*) and Becherer et al. (*2*). Oligonucleotides were synthesized and cartridge purified by Biomers (https://www.biomers.net) (Appendix Table 1).

LAMP Primer Sets

The MD LAMP included 6 primers per target, as described by Nagamine et al. (*5*), and an MD probe comprised of LB_Medc or LF_Medc, a Med, and a UR (*2*). We list oligonucleotides and UR sequences in Appendix Table 1. Standard concentration (1×) of primers, Med probes, and UR for TPHD-LAMP is 1.6 µmol/L of each forward inner primer (FIP) and backward inner primer (BIP), 0.6 µmol/L LF and 0.2 µmol/L LF_Medc, or 0.6 µmol/L LB and 0.2 µmol/L LB_Medc, 0.2 µmol/L of each F3 and B3, 0.1 µmol/L Med, and 0.05 µmol of UR. We used the same 6-primer set for singleplex LAMP assays, which contained 1.6 µmol/L of each FIP and BIP, 0.8 µmol/L of each LF and LB, and 0.2 µmol/L of each F3 and B3. We performed the singleplex LAMP assays using the intercalating dye SYBR green.

Primer Titration for TPHD-LAMP

To simultaneously amplify 2 targets in 1 reaction vessel successfully, both primer sets must perform equally. In a biplex LAMP reaction with an unbalanced assay, the more efficient assay will inhibit the amplification of the second target partially by binding the polymerase to the amplicon (*3*). Experiments using the standard 1× concentration of primers, mediator, and UR showed that the amplification of *H. ducreyi* is faster than the amplification of *T. pallidum* (data not shown). Consequently, the assay efficiency for *H. ducreyi* amplification is higher and might influence the amplification of *T. pallidum*. We also performed target titration and found that low *T. pallidum* concentrations, 3×10^3 copies/reaction, combined with high *H. ducreyi* concentrations, 3×10^5 copies/reaction, led to false-negative signals for *T. pallidum* (Appendix Table 2).

To solve this problem, we optimized the primer, Med, and UR concentrations used in TPHD-LAMP to focus on sensitivity and time to positive (t_p) and increase the support of the amplification of *T. pallidum*. We increased the concentration of primers, Med, and UR of the *T. pallidum* component to 1.25× and 1.5× and left the concentrations for the *H. ducreyi* component at the standard 1× concentration per reaction. Both concentrations for *T. pallidum* component showed better results; the concentration of 1.25× demonstrated best results and t_p. When we combined 3×10^3 copies/reaction of *T. pallidum* with 3×10^5 copies/reaction of *H. ducreyi*, we could still detect positive signals for both targets (data not shown). We fixed 1.25× concentration for *T. pallidum* component and 1× concentration for *H. ducreyi* component of the TPHD LAMP and used these for the subsequent testing of clinical samples (Appendix Table 3).

Plasmid Design

To determine analytical performance parameters of the assay and primer optimization, we obtained plasmid targets from Eurofins Scientific (https://www.eurofins.com) to use as quantified standard. Plasmids contained 300 bp of a defined conserved region of the 16S gene for *H. ducreyi* and the *polA* gene for *T. pallidum*. We diluted plasmid DNA in 10 mM Tris (pH 8) to adjust concentrations.

Clinical Performance of the TPHD-LAMP

Reference Assays

We performed a TaqMan real-time PCR targeting a 67-bp fragment of the *T. pallidum* polymerase I (*polA*) gene using previously described primers and probes (*7*). We used a plasmid containing the amplified fragment of the *polA* gene as a quantification standard covering the range 10^1–10^6 gene copies, but modified the reaction mix. In brief, the reaction encompassed 10 µL TaqMan Universal Master Mix II without Uracil-N glycosylase (Applied Biosystems, https://www.thermofisher.com) and 1.8 µL each of 10 µmol/L primer and the hydrolysis probe. We completed the reaction with 1 µL of the genomic DNA sample, independent of the DNA concentration. We used molecular-grade water to adjust the reaction volume to 20 µL and used the following cycling conditions: 50°C for 2 m, 95°C for 10 m, then 40 cycles each at 95°C for 15 s and 60°C for 60 s.

We retrieved ortholog sequence data of the *Haemophilus* 16S rRNA gene from GenBank (Appendix Figure 1, Appendix Table 4) and aligned the genes by using Geneious R11 (https://www.geneious.com). We used V-Xtractor (http://www.cmde.science.ubc.ca/mohn/software.html), a Perl-based high-throughput software tool, to locate the hypervariable regions of the 16S rRNA sequences using the Hidden Marcov Models option. In silico, we searched for regions that discriminate *H. ducreyi* from ortholog 16S rRNA gene sequences. We found suitable target sequences in the V8 region of the 16S rRNA gene and designed primers to target that region. Prior to use in the qPCR, we ran a PCR using the newly designed sense primer 5′-TAT ACA GAG GGC GGC AAA CC and the antisense primer 5′-CCA ATC CGG ACT TAG ACG TAC. Sanger sequencing of the 66-bp product confirmed the amplification of the targeted sequence of the 16S rRNA gene of *H. ducreyi*. We cloned the

product and used it to generate a plasmid quantification standard for the qPCR covering a 10^1–10^6 gene copies. Subsequently, we designed a hydrolysis probe FAM-5′ CAA AGG GGA GCG AAT CTC AC-TAMRA and used it to perform the TaqMan qPCR using the same reaction mix and cycling conditions as described for the *polA* qPCR.

LAMP and qPCR assays used the same DNA extracts and included appropriate negative controls. Because of sample restrictions, we analyzed samples as duplicates. All reactions of the *polA* and *H. ducreyi* qPCR were run on a StepOnePlus Real-Time PCR System (Applied Biosystems). We analyzed raw data by using the StepOne Software version 2.3 (Life Technologies, https://www.thermofisher.com). We considered positive reactions to be those with exponential increase of delta-Rn, a value that corresponds to the intensity of fluorescence. We excluded samples that increased in fluorescence above the threshold but failed exponential increase.

Validation of TPHD-LAMP in Clinical Samples

To validate the TPHD-LAMP, we used clinical samples to determine the sensitivity and specificity for *T. pallidum* and *H. ducreyi*, which we calculated by using the following formulas:

% Sensitivity = $\frac{\mathrm{TP}}{\mathrm{TP+FN}} \times 100$

% Specificity = $\frac{\mathrm{TN}}{\mathrm{TN+FP}} \times 100$

Positive predictive value (%) = $\frac{\mathrm{TP}}{\mathrm{TP+FP}} \times 100$

Negative predictive value (%) = $\frac{\mathrm{TN}}{\mathrm{TN+FN}} \times 100$

where TP (true positive) means positive results were confirmed with PCR; TN (true negative) means negative results were confirmed with PCR; FP (false positive) means PCR results were negative; and FN (false negative) means PCR results were positive. We calculated positive predictive values and negative predictive values for TP, TN, FP, and FN of TPHD-LAMP of clinical samples (Appendix Table 5) and for singleplex LAMP assays of clinical samples (Appendix Table 6).

Assessment of Analytical Performance

Analytical Sensitivity and Specificity

We described how we calculated values for the limit of detection (LOD) of the TPHD-LAMP in the main article (Appendix Figure 2). We determined the linearity of the biplex assays, containing both targets, was R^2(*H. ducreyi*) = 0.97 and R^2(*T. pallidum*) = 0.95.

We generated analytical specificity data by in silico analysis. Then we tested a panel including *Escherichia coli*, *Klebsiella pneumoniae*, *Acinetobacter baumannii*, *Pseudomonas aeruginosa*, *Enterobacter cloacae*, *Salmonella enterica* (Paratyphi and Typhi), *Streptococcus pneumoniae*, *Streptococcus pyogenes*, *Staphylococcus aureus*, *Corynebacterium diphtheria*, *Corynebacterium ulcerans*, *Proteus mirabilis*, and *Enterococcus faecalis*. We tested the specificity of primer sets for the single *T. pallidum* and *H. ducreyi* components of the TPHD-LAMP, and for TPHD-LAMP assay. In all cases, assays were negative within 60 m for single components (data not shown) and for TPHD-LAMP (Appendix Figure 3), demonstrating high analytical specificity.

Interassay and Intraassay Variability

Interassay variability describes reproducibility and intraassay variability describes repeatability of assays. We calculated interassay and intraassay variability of the TPHD-LAMP assay by using 3 batches of biplex LAMP mix, individually prepared on 3 separate days, processed in different runs, and ran each batch in 3 replicates. First, we evaluated TPHD-LAMP assays that contained 3×10^4 copies/reaction of a single target; then we tested them for simultaneous detection of both targets in the sample. We determined t_p as the time of the maximum increase of fluorescence, calculated by the first derivative of the fluorescence intensity, as previously described (*2*,*6*). We calculated the SD for t_p by the scattering of measurement values between triplicates.

For assays containing a single target, the TPHD-LAMP interassay coefficients of variation (CVs) were 0.9 % for *H. ducreyi* and 2.6 % for *T. pallidum*. The intraassay CVs were 0 for *H. ducreyi* and 3.5 % for *T. pallidum*. For assays containing both pathogens, the interassay CVs were 0.2 % for *H. ducreyi* and 2.8 % for *T. pallidum* and the intraassay CVs were 0.5 % for *H. ducreyi* and 2.0 % for *T. pallidum*. The t_p for *H. ducreyi* were almost constant around 7 m

independent of the presence of *T. pallidum* (Appendix Figure 4). In contrast, t_p for *T. pallidum* increased from 11.4 m ±0.4 m in assays without *H. ducreyi* to 30.4 m ± 0.8 m for TPHD-LAMP in assays containing both targets.

References

1. Knauf S, Lüert S, Šmajs D, Strouhal M, Chuma IS, Frischmann S, et al. Gene target selection for loop-mediated isothermal amplification for rapid discrimination of *Treponema pallidum* subspecies. PLoS Negl Trop Dis. 2018;12:e0006396. PubMed https://doi.org/10.1371/journal.pntd.0006396

2. Becherer L, Bakheit M, Frischmann S, Stinco S, Borst N, Zengerle R, et al. Simplified real-time multiplex detection of loop-mediated isothermal amplification using novel mediator displacement probes with universal reporters. Anal Chem. 2018;90:4741–8. PubMed https://doi.org/10.1021/acs.analchem.7b05371

3. SantaLucia J Jr. Physical principles and visual-OMP software for optimal PCR design. Methods Mol Biol. 2007;402:33–4. PubMed https://doi.org/10.1007/978-1-59745-528-2_1

4. Lehnert M, Kipf E, Schlenker F, Borst N, Zengerle R, von Stetten F. Fluorescence signal-to-noise optimisation for real-time PCR using universal reporter oligonucleotides. Anal Methods. 2018;10:3444–54. https://doi.org/10.1039/C8AY00812D

5. Nagamine K, Hase T, Notomi T. Accelerated reaction by loop-mediated isothermal amplification using loop primers. Mol Cell Probes. 2002;16:223–9. PubMed https://doi.org/10.1006/mcpr.2002.0415

6. Martineau RL, Murray SA, Ci S, Gao W, Chao S-H, Meldrum DR. Improved performance of loop-mediated isothermal amplification assays via swarm priming. Anal Chem. 2017;89:625–32. PubMed https://doi.org/10.1021/acs.analchem.6b02578

7. Chen C-Y, Chi K-H, George RW, Cox DL, Srivastava A, Rui Silva M, et al. Diagnosis of gastric syphilis by direct immunofluorescence staining and real-time PCR testing. J Clin Microbiol. 2006;44:3452–6. PubMed https://doi.org/10.1128/JCM.00721-06

Appendix Table 1. Sequences of primers, mediator displacement probes, and universal reporters for loop-mediated isothermal amplification for *Treponema* pallidum and *Haemophilus ducreyi**

Target, description	Sequence, 5′→3′	Reference
Haemophilus ducreyi		
F3	ATGTTGGGTTAAGTCCCGC	This work
B3	TCCAATCCGGACTTAGACGT	This work
FIP	CATCCCCACCTTCCTCCAGTTTTTATCCTTTGTTGCCAGCATG	This work
BIP	CATGGCCCTTACGAGTAGGGCCCCCTTTGCAGGTTTGCC	This work
LF	GCAGTCTCCTTTGAGTTCCCA	This work
LB	TACACACGTGCTACAATGGCg	This work
LB_Medc$_1$	GGTCGTAGAGCCCAGAACGAGATGAGTGGTACACACGTgcTACAATGGCg	This work
Med$_1$	CCACTCATC**TCGTTCTGGGCTCTACGACC**	This work
UR$_1$	BMN-Q-535-ATTGCGGGAGATGAGACCCGCAA-dTFAM-TGTT**GGTCGTAGAGCCCAGAACGA**-C3	(*2*,*6*)
Treponema pallidum		
F3	GATTGGTCCTAAGACGGC	This work
B3	GGAATACAACAGGAATCTTCGA	This work
FIP	CAGCGCTTCTTTTAAGGAATAGGTATGCACATCTTCTCCACTG	This work
BIP	TGTGGGAAGAAAGATGCATTTTTTTAAAAAACACATGGTACATCGT	This work
LF	CGATAAATACCATCAAGTGTGCCA	This work
LB	CGTTCACTCATTGAGTTGCGTG	This work
LF_Medc$_2$	CACTGACCGAACTGAGCTCCTGAGGCATGGTTTTCGATAAATACCATCAAGTGTGCCA	This work
Med$_2$	CCATGCCTCAGGAGCT**CAGTTCGGTCAGTG**	(*2*)
UR$_2$	BMN-Q-535-CACCGGCCAAGACGCGCCGG-dT-Atto-647N-GTGTT**CACTGACCGAACTG**GAGCA-C3	(*2*,*6*)

*Underlined sequences illustrate complementary regions between LF/LB_Medc and mediator (Med). Bold text indicates nucleotides in complementary regions of mediator and universal reporters. Indices 1 and 2 indicate complementary sequences in Med and Medc or UR. BIP, backward inner primer; FIP, forward inner primer; LB, Loop B; LF, Loop F; UR, universal reporter.

Appendix Table 2. Positive results for *Treponema pallidum* and *Haemophilus ducreyi* in 3 TPHD-LAMP assays before primer titration*

T. pallidum, concentration	*H. ducreyi*, concentration	No. *H. ducreyi*–positive	No. *T. pallidum*–positive
3×10^3 copies/reaction	3×10^5 copies/reaction	3	0
3×10^5 copies/reaction	3×10^5 copies/reaction	3	3
3×10^5 copies/reaction	3×10^3 copies/reaction	3	3
3×10^3 copies/reaction	3×10^3 copies/reaction	3	3
3×10^4 copies/reaction	3×10^4 copies/reaction	3	3
0	0	0	0

*Positive results reflect number of positive results in 3 reactions at each combined concentration of *Treponema pallidum* and *Haemophilus ducreyi* loop-mediated isothermal amplification (LAMP).

Appendix Table 3. Primer composition for the biplex loop-mediated isothermal amplification of *Treponema pallidum* and *Haemophilus ducreyi* for the clinical validation.

Target	Oligonucleotide	Concentration, µmol/L
H. ducreyi	F3	0.20
	B3	0.20
	FIP	1.60
	BIP	1.60
	LF	0.80
	LB	0.60
	LB _Medc$_1$	0.20
	Med$_1$	0.10
T. pallidum	F3	0.25
	B3	0.25
	FIP	2.00
	BIP	2.00
	LF	0.75
	LB	1.00
	LF _Medc$_2$	0.25
	Med$_2$	0.13

Appendix Table 4. Ortholog sequence data of the *Haemophilus* 16S rRNA gene retrieved from GenBank and used for the *H. ducreyi* TaqMan qPCR design

GenBank accession no.	Pathogen	Strain no.
DQ851143_1	*Haemophilus simiae*	ROG53
AF224307_1	*Haemophilus quentini*	NA
CP008740_1	*Haemophilus influenzae*	2019
CP008740_1_2	*Haemophilus influenzae*	2019
AY613457_1	*Haemophilus influenzae*	M8943
AY613451_1	*Haemophilus influenzae*	M9741
CP007470_1_6	*Haemophilus influenzae*	477
CP007470_1_5	*Haemophilus influenzae*	477
CP007470_1_4	*Haemophilus influenzae*	477
CP007470_1_3	*Haemophilus influenzae*	477
CP007470_1	*Haemophilus influenzae*	477
CP007470_1_2	*Haemophilus influenzae*	477
EF399173_1	*Unclutured bacterium*	SJTU_F_12_59
AB597550_1	*Unclutured gamma proteobacterium*	NA
AB597543_1	*Unclutured gamma proteobacterium*	NA
AB597538_1	*Unclutured gamma proteobacterium*	NA
FQ312002_1_6	*Haemophilus parainfluenza*	T3T1
FQ312002_1_2	*Haemophilus parainfluenza*	T3T1
FQ312002_1	*Haemophilus parainfluenza*	T3T1
FQ312002_1_5	*Haemophilus parainfluenza*	T3T1
CP006956_1	*Bibersteinia trehalosi*	USDA-ARS-USMARC-190
CP006956_1_2	*Bibersteinia trehalosi*	USDA-ARS-USMARC-190
CP006956_1_6	*Bibersteinia trehalosi*	USDA-ARS-USMARC-190
CP006955_1_4	*Bibersteinia trehalosi*	USDA-ARS-USMARC-189
CP003745_1_3	*Bibersteinia trehalosi*	USDA-ARS-USMARC-192
CP006955_1_2	*Bibersteinia trehalosi*	USDA-ARS-USMARC-189
CP006955_1	*Bibersteinia trehalosi*	USDA-ARS-USMARC-189
CP003745_1	*Bibersteinia trehalosi*	USDA-ARS-USMARC-192
CP006944_1	*Mannheimia varigena*	USDA-ARS-USMARC-1312
CP006944_1_5	*Mannheimia varigena*	USDA-ARS-USMARC-1312
CP006953_1_3	*Mannheimia varigena*	USDA-ARS-USMARC-1388
CP006953_1	*Mannheimia varigena*	USDA-ARS-USMARC-1388
CP006943_1_2	*Mannheimia varigena*	USDA-ARS-USMARC-1296
CP006943_1_5	*Mannheimia varigena*	USDA-ARS-USMARC-1296
CP006943_1_6	*Mannheimia varigena*	USDA-ARS-USMARC-1296
CP006943_1_3	*Mannheimia varigena*	USDA-ARS-USMARC-1296
LN795822_1	*Mannheimia sp.*	MG13
AF053895_1	*Mannheimia sp.*	HPA102
AF053890_1	*Mannhaimia glucosida*	UT18
KU051693.1	*Mannheimia haemolytica*	A2
CP011099_1_6	*Mannheimia haemolytica*	89010807N
CP004753_2_6	*Mannheimia haemolytica*	USDA-ARS-USMARC-185
CP005972_1_6	*Mannheimia haemolytica*	D153
CP023044_1_4	*Mannheimia haemolytica*	191
CP006574_1_4	*Mannheimia haemolytica*	D174
CP023043_1_2	*Mannheimia haemolytica*	193
CP005972_1	*Mannheimia haemolytica*	D153
DQ301920_1	*Mannheimia haemolytica*	PHL213
CP005383_1_6	*Mannheimia haemolytica*	M42548
CP006957_2_6	*Mannheimia haemolytica*	USDA-ARS-USMARC-184
CP006957_2_4	*Mannheimia haemolytica*	USDA-ARS-USMARC-184
CP006957_2_2	*Mannheimia haemolytica*	USDA-ARS-USMARC-184
GQ358868_1	*Uncultured bacterium*	clone 8837-D0-O-7D
M75079_1	*Haemophilus ducreyi*	35000
M75084_1	*Haemophilus ducreyi*	KC57
M75078_1	*Haemophilus ducreyi*	CPI 542
CP015434_1_5	*Haemophilus ducreyi*	GHA9
CP015434_1	*Haemophilus ducreyi*	GHA9
AE017143_1_2	*Haemophilus ducreyi*	35000
ST16SrRNA_3_1490596	*Haemophilus ducreyi*	1490596
AF525028_1	*Haemophilus ducreyi*	isolate Amsterdam
CP015432_1	*Haemophilus ducreyi*	GHA5
CP015426_1_2	*Haemophilus ducreyi*	VAN3
NR_044741_1	*Haemophilus ducreyi*	CIP 54.2
CP015425_1_2	*Haemophilus ducreyi*	VAN2
NZ_CP015429	*Haemophilus ducreyi*	GHA1
CP015430_1_5	*Haemophilus ducreyi*	GHA2

GenBank accession no.	Pathogen	Strain no.
CP015430_1_6	*Haemophilus ducreyi*	GHA2
CP015430_1	*Haemophilus ducreyi*	GHA2
AY513483_1	*Haemophilus ducreyi*	ATCC 33921
HE681373_1	*Uncultured bacterium*	clone 7q_13
AY005034_1	*Haemophilus sp.*	clone BJ021
AF224283_1	*Actinobacillus pleuropneumoniae*	MCCM 00189
LK985384_1	*Haemophilus parahaemolyticus*	isolate G321
AF224285_1	*Actinobacillus capsulatus*	CCUG 37035
CP009158_1_6	*Haemophilus parasuis*	SH03
CP005384_1	*Haemophilus parasuis*	ZJ0906
CP001321_1	*Haemophilus parasuis*	SH0165
CP001321_1_6	*Haemophilus parasuis*	SH0165
CP001321_1_4	*Haemophilus parasuis*	SH0165
CP001321_1_5	*Haemophilus parasuis*	SH0165
CP001321_1_3	*Haemophilus parasuis*	SH0165
CP005384_1_3	*Haemophilus parasuis*	ZJ0906
CP005384_1_4	*Haemophilus parasuis*	ZJ0906
CP005384_1_5	*Haemophilus parasuis*	ZJ0906
CP020085_1_5	*Haemophilus parasuis*	CL120103
CP015099_1_5	*Haemophilus parasuis*	SC1401
CP020085_1_6	*Haemophilus parasuis*	CL120103
CP015099_1_2	*Haemophilus parasuis*	SC1401
CP015099_1	*Haemophilus parasuis*	SC1401
CP020085_1_4	*Haemophilus parasuis*	CL120103
CP020085_1	*Haemophilus parasuis*	CL120103
AB559648_1	*Uncultured bacterium*	clone c_GA_H2
AF371853_1	*Uncultured bacterium*	clone bp-2130-s959–2
DQ926692_1	*Actinobacillus porcitonsillarum*	73706
DQ926691_1	*Actinobacillus porcitonsillarum*	71123
DQ381154_1	*Pasteurella caballi*	NSVL 84679
AF224291_1	*Pasteurella caballi*	MCCM 00841
HF565184_1	*Actinobacillus sp.*	MK-2012
HF565188_1	*Actinobacillus sp.*	MK-2012
HF565186_1	*Actinobacillus sp.*	MK-2012
KC834743_1	*Actinobacillus pleuropneumoniae*	TJ12
KC834744_1	*Actinobacillus pleuropneumoniae*	HB13
NR_115546_2	*Actinobacillus pleuropneumoniae*	Shope 4074
D30030_1	*Actinobacillus pleuropneumoniae*	NA
CP022715_1_2	*Actinobacillus pleuropneumoniae*	KL 16
CP022715_1_4	*Actinobacillus pleuropneumoniae*	KL 16
CP000569_1_3	*Actinobacillus pleuropneumoniae*	L20 serotype 5b
CP000569_1_2	*Actinobacillus pleuropneumoniae*	L20 serotype 5b
CP000569_1	*Actinobacillus pleuropneumoniae*	L20 serotype 5b
CP001091_1_6	*Actinobacillus pleuropneumoniae*	serotype 7, str. AP76
CP000687_1_6	*Actinobacillus pleuropneumoniae*	serotype 3, str. JL03
CP000687_1_5	*Actinobacillus pleuropneumoniae*	serotype 3, str. JL03
CP001091_1_4	*Actinobacillus pleuropneumoniae*	serotype 7, str. AP76
D30032_1	*Actinobacillus pleuropneumoniae*	NA
D30031_1	*Actinobacillus pleuropneumoniae*	NA
AY749139_1	*Actinobacillus genomo sp.*	52418–03
AY749138_1	*Actinobacillus genomo sp.*	52418–03
AF247722_1	*Actinobacillus lignieresii*	F 127
AY749137_1	*Actinobacillus genomo sp.*	24593–01
AF247723_1	*Actinobacillus lignieresii*	F 264
CP003875_1_6	*Actinobacillus suis*	H91–0380
LT906456_1	*Actinobacillus suis*	NCTC12996
LT906456_1_3	*Actinobacillus suis*	NCTC12996
CP009159_1_6	*Actinobacillus suis*	ATCC 33415
CP007715_1_4	*Actinobacillus equuli subsp. equuli*	19392
CP007715_1_3	*Actinobacillus equuli subsp. equuli*	19392
AY749144_1	*Actinobacillus equuli subsp. haemolyticus*	27368–01
AY749142_1	*Actinobacillus equuli subsp. haemolyticus*	23611–01
AY749141_1	*Actinobacillus equuli subsp. haemolyticus*	23596–01
CP007715_1_2	*Actinobacillus equuli subsp. equuli*	19392
AY749140_1	*Actinobacillus equuli subsp. haemolyticus*	23337–01

Appendix Table 5. Sensitivity and specificity of biplex loop-mediated isothermal amplification of *Treponema pallidum* and *Haemophilus ducreyi* in samples from 293 patients with suspected *T. pallidum* infection

Characteristics	Sample size, no.	*Treponema pallidum*, no.	*Haemophilus ducreyi*, no.
All samples	293		
True positive		50	142
True negative		224	116
False positive		10	21
False negative		9	13
Positive predictive value, %		83.3	87.1
Negative predictive value, %		96.1	89.9
Lihir Island	57		
True positive		19	13
True negative		34	39
False positive		2	0
False negative		2	4
Positive predictive value, %		90.5	100
Negative predictive value, %		94.4	90.7
Karkar Island	184		
True positive		25	99
True negative		144	59
False positive		8	20
False negative		7	6
Positive predictive value, %		75.8	83.2
Negative predictive value, %		95.4	90.8
Ghana	52		
True positive		6	30
True negative		46	18
False positive		0	1
False negative		0	3
Positive predictive value, %		100	96.8
Negative predictive value, %		100	85.7

Appendix Table 6. Sensitivity and specificity of singleplex loop-mediated isothermal amplification for *Treponema pallidum* and *Haemophilus ducreyi* in samples from 293 patients with suspected *T. pallidum* infection

Results	*Treponema pallidum*, no.	*Haemophilus ducreyi*, no.
True positive	46	141
True negative	229	103
False positive	5	34
False negative	13	14
Positive predictive value, %	90.2	80.6
Negative predictive value, %	94.6	88.0

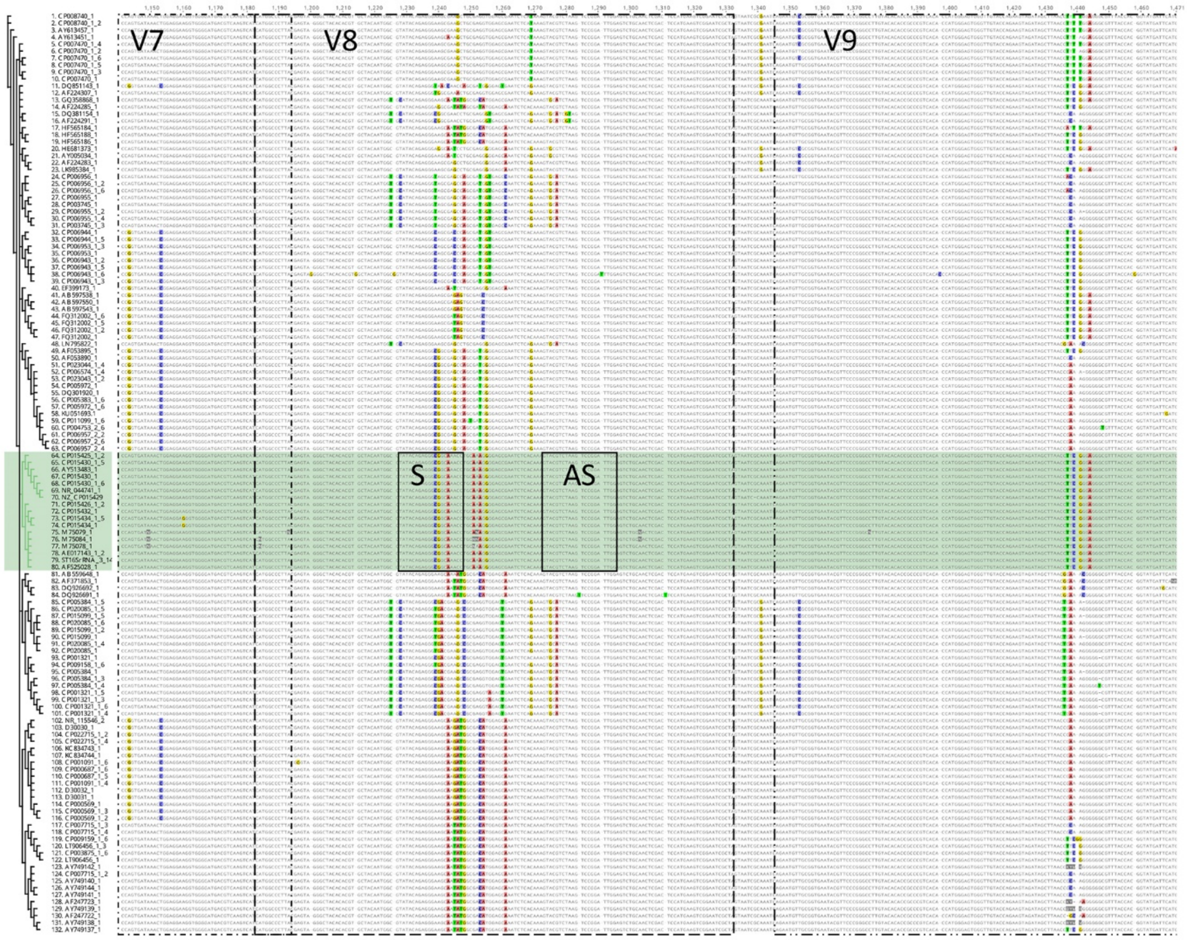

Appendix Figure 1. Nucleotide sequence identity chart for variable regions 7–9 and dendrogram for variable regions 1–9 of orthologue sequence data of the 16S rRNA gene of *Haemophilus ducreyi*. Sequence differences are highlighted. The region inside the dotted line V7 represents variable region 7, the region inside dotted line V8 represents variable region 8, and the region inside dotted line V9 represents the variable region 9 of the 16S rRNA gene. Sequences in green band denote *H. ducreyi* specific sequences. The S-box indicates the binding region of sense primer and the AS-box indicates the binding region of the antisense primer for the optimized qPCR for *H. ducreyi*. Sequences were aligned using Geneious (https://www.geneious.com). GenBank accession numbers correspond to the data shown in Appendix Table 4.

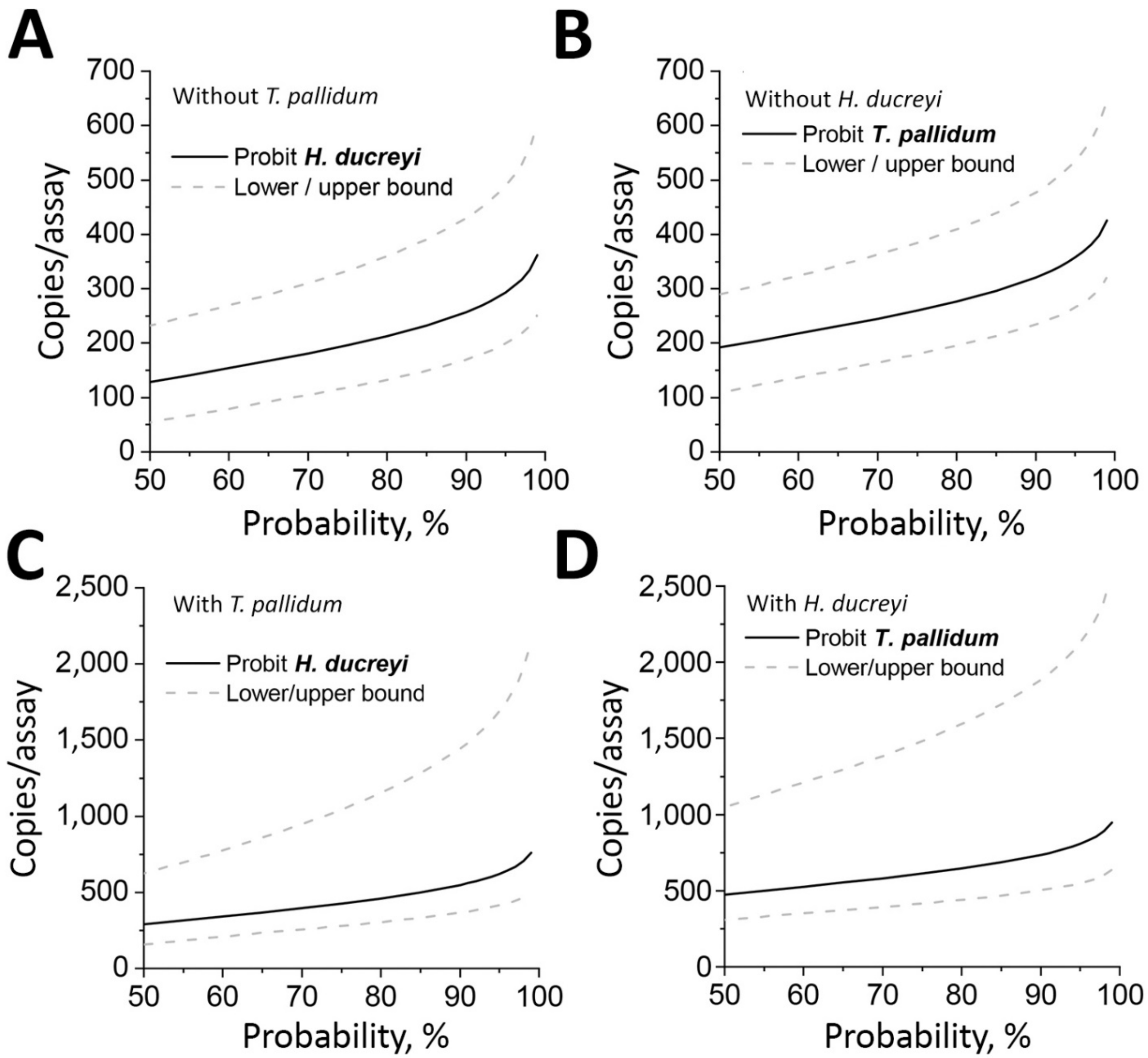

Appendix Figure 2. Limit of detection (LOD) for *Treponema pallidum* and *Haemophilus ducreyi* loop-mediated isothermal amplification (TPHD-LAMP) assay. The probability of successful amplification of a given copy number was predicted using Probit analysis. Lower and upper bounds, illustrated as dashed lines, represent 95% CI. A) LOD for *H. ducreyi* in samples without *T. pallidum*. B) LOD for *T. pallidum* in samples without *H. ducreyi*. C) LOD for *H. ducreyi* in the presence of 3×10^5 copies *T. pallidum* plasmid DNA. D) LOD for *T. pallidum* in the presence of 3×10^5 copies *H. ducreyi* plasmid DNA.

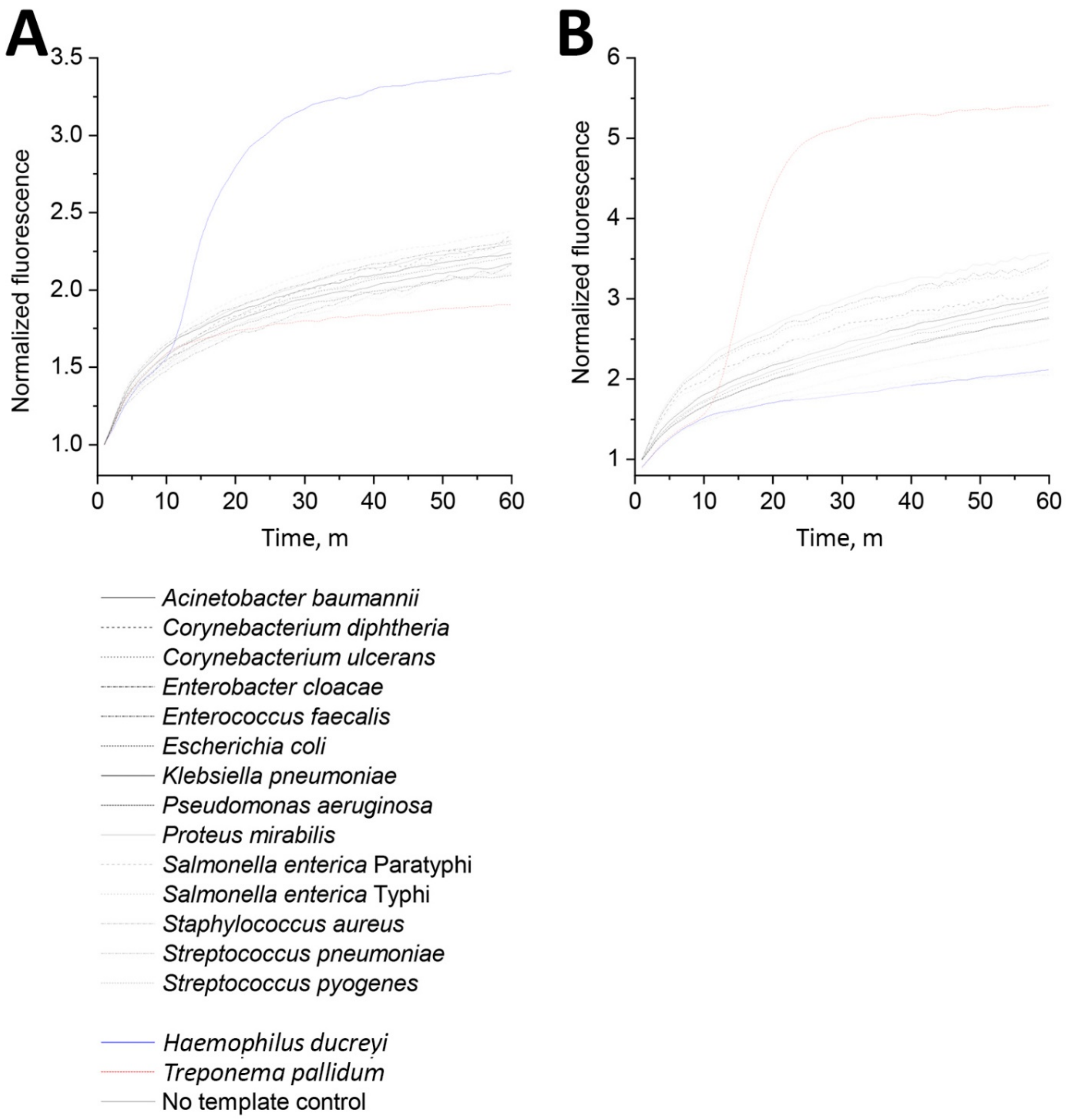

Appendix Figure 3. Analytical specificity of *Treponema pallidum* and *Haemophilus ducreyi* loop-mediated isothermal amplification (TPHD-LAMP) assay against pathogens other than *T. pallidum* and *H. ducreyi*. Specificity of TPHD-LAMP for A) *H. ducreyi* in mixed samples; B) *T. pallidum* in mixed samples.

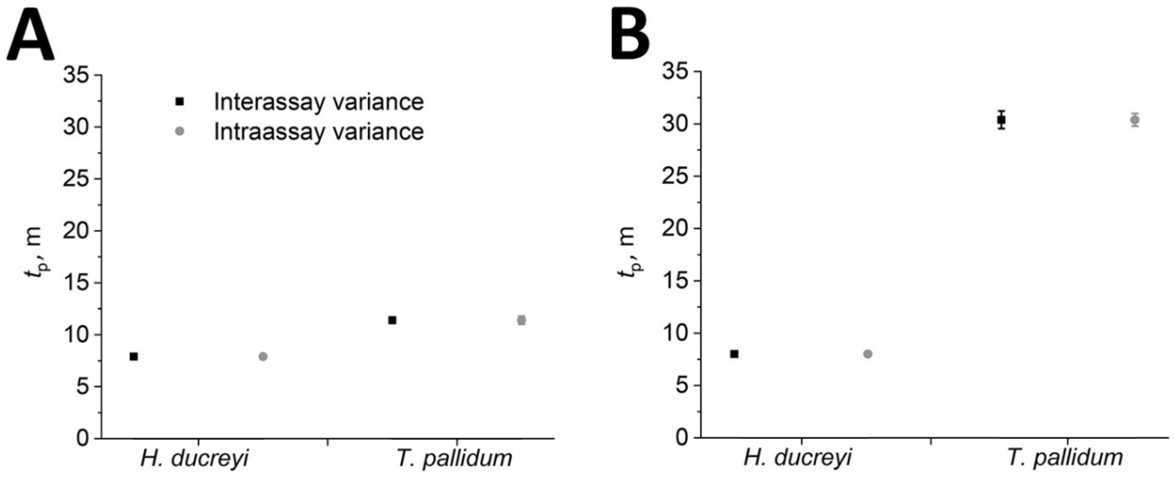

Appendix Figure 4. Interassay and intraassay variance of *Treponema pallidum* and *Haemophilus ducreyi* loop-mediated isothermal amplification (TPHD-LAMP) assay illustrated for the amplification of 1 target and of both targets in the sample. The mean value of time to positive (t_p) is plotted against target and type of variability. The variability is illustrated by error bars, which reflect SD values. A) Variance in the presence of 3×10^4 copies/reaction of 1 target, either *T. pallidum* or *H. ducreyi* in the sample. B) Variance in the presence of 3×10^4 copies/reaction of *T. pallidum* and 3×10^4 copies/reaction *H. ducreyi* in the sample.
